# Direct Growth of III-Nitride Nanowire-Based Yellow Light-Emitting Diode on Amorphous Quartz Using Thin Ti Interlayer

**DOI:** 10.1186/s11671-018-2453-1

**Published:** 2018-02-06

**Authors:** Aditya Prabaswara, Jung-Wook Min, Chao Zhao, Bilal Janjua, Daliang Zhang, Abdulrahman M. Albadri, Ahmed Y. Alyamani, Tien Khee Ng, Boon S. Ooi

**Affiliations:** 1 Photonics Laboratory, King Abdullah University of Science and Technology (KAUST), Thuwal, 23955-6900 Saudi Arabia; 2 Imaging and Characterization Core Laboratory, King Abdullah University of Science and Technology (KAUST), Thuwal, 23955-6900 Saudi Arabia; 3 National Center for Nanotechnology, King Abdulaziz City for Science and Technology (KACST), Riyadh, 11442-6086 Saudi Arabia

**Keywords:** Nanowires, True yellow, Gallium nitride, Amorphous quartz, Light-emitting diodes

## Abstract

Consumer electronics have increasingly relied on ultra-thin glass screen due to its transparency, scalability, and cost. In particular, display technology relies on integrating light-emitting diodes with display panel as a source for backlighting. In this study, we undertook the challenge of integrating light emitters onto amorphous quartz by demonstrating the direct growth and fabrication of a III-nitride nanowire-based light-emitting diode. The proof-of-concept device exhibits a low turn-on voltage of 2.6 V, on an amorphous quartz substrate. We achieved ~ 40% transparency across the visible wavelength while maintaining electrical conductivity by employing a TiN/Ti interlayer on quartz as a translucent conducting layer. The nanowire-on-quartz LED emits a broad linewidth spectrum of light centered at true yellow color (~ 590 nm), an important wavelength bridging the green-gap in solid-state lighting technology, with significantly less strain and dislocations compared to conventional planar quantum well nitride structures. Our endeavor highlighted the feasibility of fabricating III-nitride optoelectronic device on a scalable amorphous substrate through facile growth and fabrication steps. For practical demonstration, we demonstrated tunable correlated color temperature white light, leveraging on the broadly tunable nanowire spectral characteristics across red-amber-yellow color regime.

## Background

The use of light-emitting diode (LED) for display technology has become widespread over the past decade. These light sources are more energy efficient compared to cold cathode fluorescent lamp (CCFL) and more suitable for portable consumer electronics. Conventional LEDs rely on GaN-based blue LEDs grown on sapphire substrates. As the demand for LED products increases, the trend is shifting towards the use of larger diameter sapphire substrate to scale up manufacturing yield. However, the large-size sapphire substrate is challenging to manufacture due to difficulties in precise drilling of c-plane sapphire from Kyropoulos boules while maintaining accurate crystal orientation and flatness with increasing diameter [1, 2]. In addition to manufacturing issues, conventional planar GaN-based LEDs are constrained by the existence of the green-gap, i.e., the spectral region where the LED quantum efficiency decreases for wavelengths longer than the green wavelength (520 nm).

There have been several attempts to grow III-nitride materials atop glass-based substrates. Previously, epitaxial growth of GaN on glass using gas-source molecular beam epitaxy (MBE) [3] and sputtering [4, 5] have produced low-quality polycrystalline material, affecting device performance. Alternatively, Samsung has demonstrated the capability of growing nearly single-crystalline GaN pyramids on glass by micromasking and subsequent selective metal organic chemical vapor deposition (MOCVD) growth [6, 7]. However, the excessive indium evaporation in MOCVD prevents efficient incorporation of indium for achieving emitters in the green-gap. Shon et al. demonstrated the possibility of improving the quality of a sputtered InGaN thin film on amorphous glass using graphene as a pre-orienting buffer layer, effectively suppressing the defect-related photoluminescence [8]. Nevertheless, these methods require complex processing steps that hinder the potential for integration into large-scale manufacturing processes.

One possible method of directly incorporating III-nitride light emitter with glass-based substrate is by utilizing spontaneously grown III-nitride nanowires using MBE. By optimizing the growth condition, it is possible to have III-nitride nanowires grow spontaneously without the need for any templated growth-mask or catalyst [9]. Because of the large surface to volume ratio, the nanowires can grow free of threading dislocation [10] while having reduced strain in the active region. The reduced strain enables fabrication of III-nitride nanowire-based devices working within the green-gap and beyond [11–16]. The III-nitride nanowires have been shown to grow on various substrates, such as silicon [9, 17, 18], metal [19–21], and silica [22–25], making it possible to utilize a broad range of substrates. Currently, due to the insulating nature of the glass-based substrate, it is challenging to fabricate an electrically injected device atop silica while maintaining both conductivity and transparency simultaneously.

In this work, we undertook this challenge, and successfully demonstrate the growth and fabrication of an InGaN/GaN nanowire-based LED grown on an amorphous quartz substrate. We achieved simultaneous transparency and conductivity by employing a translucent TiN/Ti interlayer as the conductive layer and the growth site for the nanowires. Because the nanowires grow spontaneously without a requisite global epitaxial relationship with the substrate, no complex or expensive processing steps are required before material growth. The nanowires-on-quartz LED emits a broad linewidth yellow light centered at ~ 590 nm, a color that is challenging to achieve with conventional planar quantum-well nitride technologies, thus further accentuate the significance of our current work.

For a practical demonstration, we have also performed correlated color temperature (CCT) tuning experiment based on mixed spontaneous and stimulated light sources. The use of transparent amorphous quartz allows the direct transmission of light from a laser diode for white light generation. Growing nanowires on quartz opens up new possibilities and opportunities for realizing integrated light emitters operating in the green-gap while benefiting from the scalability of amorphous quartz technology. Despite the technological infancy as compared to planar group III-nitride LEDs, the unique properties of nitrided titanium for nanowire growth is paramount to enabling seamless integration of light emitter on transparent substrate.

## Methods

### Material Growth

The nanowires-on-quartz samples were grown catalyst-free under nitrogen-rich condition using a Veeco GEN 930 PA-MBE system. A commercial double polished amorphous quartz substrate (thickness ~  500 *μ* m) is first cleaned using acetone and isopropyl alcohol rinse and dried using nitrogen blow-dry. Before growth, 20-nm thick Ti layer was deposited using electron beam evaporation to act as the translucent conducting interlayer. After Ti deposition, another round of solvent cleaning using acetone and isopropyl alcohol is performed. Two rounds of outgassing were performed to remove any moisture and contaminants from the substrate surface. After loading into the growth chamber, the substrate surface is exposed to a nitrogen plasma to partially convert the Ti into TiN before opening the Ga shutter. The nitrogen was kept at 1-sccm flow rate and 350-W RF power during nitridation and throughout the growth process. For n-type GaN:Si nanowire base growth, the Ga beam equivalent pressure (BEP) was 6.5 × 10^−8^ Torr while Si cell temperature was kept at 1165 °C. We utilized a two-step growth method to obtain high-quality GaN while controlling the density of the nanowires. The GaN nanowire nucleation layer was deposited at a substrate temperature of 620 °C for 10 min, followed by GaN nanowire growth at an elevated temperature (770 °C). After n-GaN growth, the active region consisting of five pairs of InGaN quantum disks and GaN quantum barriers were deposited. In BEP was 5 × 10^−8^ Torr, and Ga was 3 × 10^−8^ Torr for quantum disk growth. A p-type GaN:Mg section was grown after the final GaN quantum barrier. The Mg cell was kept at 310 °C during p-GaN growth.

### Optical and Structural Characterization

The photoluminescence (PL) characteristics of the nanowires grown on quartz was measured using temperature-dependent *μ*-PL measurements using a 325-nm HeCd laser as the excitation source and × 15 UV objective lens. The output power of the laser is ~ 3.74 mW. The beam spot size is ~ 1.24 *μ*m, which gives a corresponding excitation power density of ~ 310 kW/cm^2^. The sample was cooled to liquid nitrogen temperature using a cryostat cell (Linkam, THMS 6000). The temperature is then adjusted from 77 to 300 K. Sample transparency was measured using a Shimadzu UV-3600 UV-vis-NIR spectrophotometer. Calibration was performed using air as the reference. SEM images were taken using FEI quanta 600. High-resolution transmission electron microscopy (HRTEM) and high-resolution high-angle annular dark field STEM (HAADF-STEM) characterizations were carried out using a Titan 80-300 ST transmission electron microscope (FEI Company) operated at an accelerating voltage of 300 kV. The elemental composition map were obtained via energy dispersive X-ray spectroscopy (EDS) from EDAX Company.

### Device Fabrication and Characterization

The device fabrication is as follows. First, the as-grown nanowire sample is cleaned through standard solvent cleaning using acetone and isopropyl alcohol followed by nitrogen blow dry. Next, ~ 2 *μ* m of parylene C is deposited through thermal evaporation. An etch-back process using oxygen plasma reactive ion etching (RIE) is performed to expose the p-type nanowire tips. Afterwards, 5 nm of Ni is deposited using electron-beam evaporation followed by 230 nm of indium tin oxide (ITO) deposited using RF magnetron sputtering as the transparent current spreading layer. Annealing is done at 500 °C under Ar ambient to improve the electrical characteristics of the Ni/ITO transparent current spreading layer. Inductively coupled plasma (ICP) RIE etching is done using Cl- and Ar-based ions to define device mesa. Finally, a Ni/Au contact pad is deposited through electron-beam evaporation followed by lift-off. L-I-V characterization was performed using a Keithley 2400 power meter. Thermal measurement and imaging was conducted using a commercial Optotherm micro radiometric thermal imaging microscope. Prior to actual temperature measurement, a 2D emissivity mapping table is constructed for each pixel of the image in order to take into account the different surface emissivity values caused by different material components. This is done by heating the device to 60 °C using a heating stage and constructing the table using the Thermalyze thermal image analysis software provided by the system. After the table is constructed, the heating stage is switched off and current-dependent measurement is performed.

## Results and Discussion

### Structural and Optical Characterization of Nanowires Grown on Quartz

**Fig. 1:**
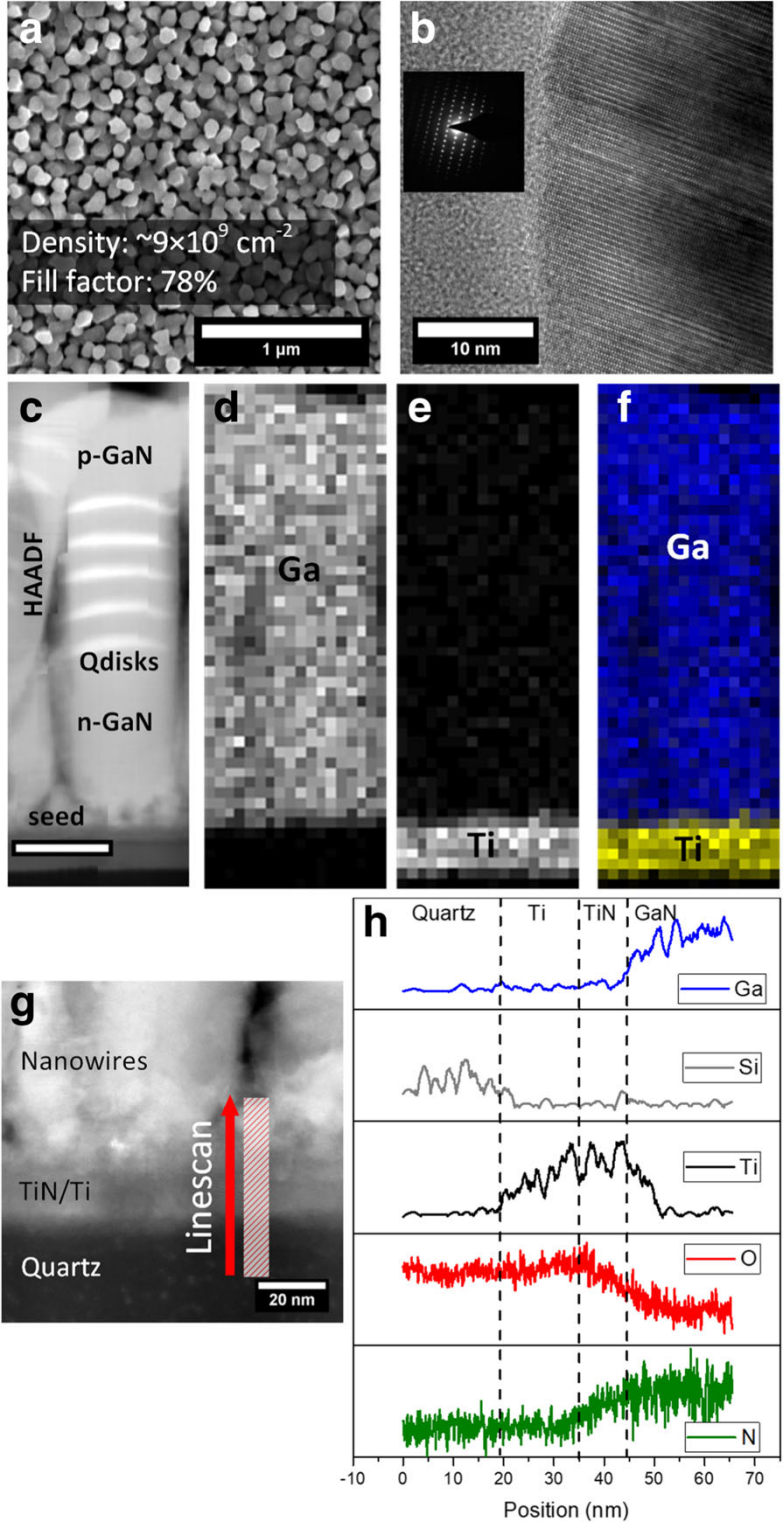
**a** Plan view SEM of as-grown InGaN/GaN nanowires grown on quartz. **b** High-magnification view of bright field TEM from the p-GaN region, showing the crystallinity of the nanowire. Inset shows the selective area electron diffraction pattern taken from the nanowire. **c** HAADF image of a single nanowire and **d** corresponding EDX map for Ga, **e** Ti, and **f** composite elemental mapping. Scale bar corresponds to 25 nm. **g** High-magnification view of the interface between the nanowire base, interlayer, and substrate. Red arrow indicates the direction for the elemental mapping. **h** Corresponding EDX and EELS results showing the change in elemental composition across the material interfaces. The EDX results are smoothed to remove noise

The nanowire structure consists of ~ 90 nm n-GaN, five pairs of ~ 7-nm thick InGaN quantum disk and ~ 14-nm thick barrier, and ~ 60 nm p-GaN. Figure 1a shows a plan-view scanning electron microscope (SEM) image of high-density nanowires. The nanowires have a typical lateral size of ~ 100 nm and length of ~ 250 nm. The density of the nanowires is calculated statistically to be ~ 9 × 10^9^ cm ^−2^, with a fill factor of 78%. Although some degree of coalescence between several nanowires can be observed, most of the nanowires appear disjointed. The growth conditions were optimized using a two-step growth method separating the initial GaN seed nucleation and nanowire growth [26]. By using this method, we were able to grow high-quality nanowire with maximum nanowire density while minimizing the coalescence between nanowires, which is detrimental to device performance because of nonradiative defects at the coalescence sites [27].

High-resolution bright field transmission electron microscope (TEM) image of the nanowire is shown in Fig. 1b, along with the corresponding selective area diffraction pattern shown in the inset. The diffraction pattern is an indication of the crystallinity of the nanowire, showing the growth high-quality GaN material on a lattice-mismatched substrate. High-angle annular dark field (HAADF) image of a single nanowire along with the corresponding elemental mapping is shown in Fig. 1c–f. The HAADF image shows five InGaN quantum disk (qdisk) insertions as the active region, indicated by brighter spots in the nanowire. At the base of the nanowire, a debris-like layer can be seen. This layer is the remains of the initial GaN nanowire seed that does not grow into nanowire due to shadowing effect. Elemental mapping shows that the nanowires grow on top of the Ti interlayer and not directly on top of the quartz substrate.

The TEM elemental mapping on the interface between the nanowire, interlayer, and quartz substrate is also shown in Fig. 1g–h to give a better understanding of the composition of the interface. Elemental mapping for Ga, Ti, and Si was performed using energy-dispersive X-ray analysis (EDX) while elemental mapping for O and N was performed using electron energy loss spectroscopy (EELS). Elemental mapping performed at the interface confirms that the top part of the Ti layer was partially converted into TiN during growth inside the MBE chamber, as indicated by the simultaneous presence of Ti and N on top of the interlayer. The TiN layer is estimated to be ~ 10 nm thick. The GaN seed nucleation and nanowire growth then occur on top of the TiN layer. EELS result shows the existence of oxygen signal across the TiN/Ti layer. This is caused by the spontaneous formation of native TiO_2_ film as the TEM sample is exposed to air after preparation [28]. The direct nucleation on TiN is advantageous for our device design, as TiN is shown to be capable of simultaneous transparency and conductivity [29] while also improving the quality of GaN grown on top of it [30] and acting as a reflector on longer wavelength [31].

**Fig. 2:**
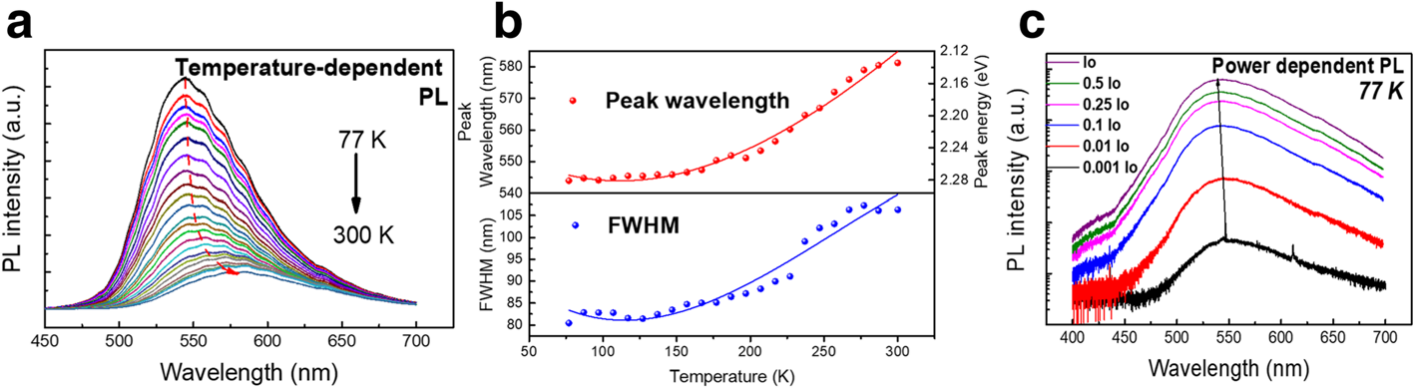
**a** Temperature dependent PL measurement result from 77 to 300 K. **b** Change of peak wavelength and FWHM for temperature-dependent PL measurement. **c** Power-dependent *μ*-PL measurement performed at 77 K, indicating reduced quantum-confined Stark effect

The optical characteristics of the nanowires grown on quartz was measured using a *μ*-PL setup with 325-nm excitation from a HeCd laser. At room temperature, *μ*-PL spectra show a broad peak. The wide linewidth is a common feature among III-nitride nanowires because of inherent structural and compositional inhomogeneity among individual nanowires [32]. The temperature-dependent *μ*-PL in Fig. 2a shows that from 77 to 300 K, *μ*-PL spectra are red-shifted and broaden with increasing temperature. The peak wavelength and FWHM for various measured temperature are shown in Fig. 2b. The red-shifting is due to Varshni effect-related temperature-dependent bandgap shrinkage, while the peak broadening with an increase of temperature is due to the coupling of excitons with acoustic phonons [33]. The reduction in peak intensity observed with increasing temperature is caused by the increase of nonradiative recombination due to the activation of nonradiative recombination centers at elevated temperature, and carriers obtaining enough thermal energy to escape the quantum disk to recombine nonradiatively. The results of the power-dependent *μ*-PL experiment at 300 K (Fig. 2c) show that the spectra exhibit negligible blue-shifting with increasing excitation power. The absence of blue-shifting can be attributed to the reduction of piezoelectric field and the quantum-confined Stark effect (QCSE) within the quantum disks caused by radial strain relaxation in nanowire structures [34].

**Fig. 3:**
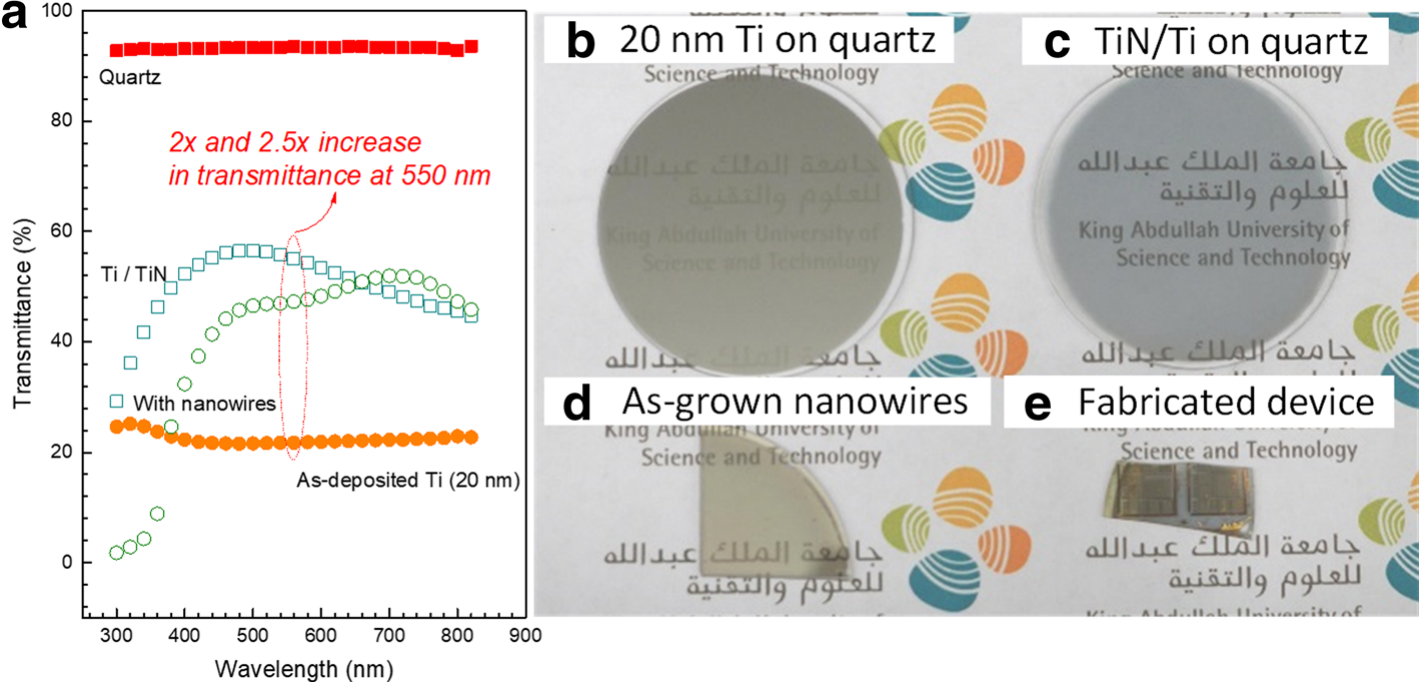
**a** Transmittance measurement results for bare quartz, quartz substrate coated with 20 nm of Ti, quartz substrate coated with TiN/Ti layer, and as-grown nanowire sample on quartz. **b** Optical photograph of quartz coated with 20 nm Ti; **c** quartz coated with Ti which has undergone partial nitridation; **d** as-grown nanowire sample; and **e** fabricated LED device on quartz

To verify the feasibility of the nanowires grown on an amorphous quartz sample for transparent device applications, we compared the transmittance of a quartz substrate coated with 20 nm of Ti, a quartz substrate coated with Ti which has undergone partial nitridation, and as-grown nanowires on quartz sample. The bare quartz substrate itself has a transmittance of ~ 93% across the visible wavelength spectrum. The measurement results are shown in Fig. 3a. For bare quartz substrate coated with 20 nm of Ti (Fig. 3b), the transmittance is only ~ 22%. After nitridation (Fig. 3c), the transmittance increases considerably by more than 20%, due to the formation of TiN layer, as confirmed by the TEM results. After nanowire growth (Fig. 3d), the transmittance is partially reduced due to light absorption from the InGaN quantum disk active region [35]. For wavelength shorter than GaN emission wavelength, the transmittance approaches zero as the GaN nanowires itself will also absorb the transmitted light. The optical photographs of quartz substrate coated with 20 nm Ti, quartz substrate with TiN/Ti layer, as-grown nanowires on quartz, and fabricated device are shown in Fig. 3b–e for comparison.

### Device Characterization

**Fig. 4:**
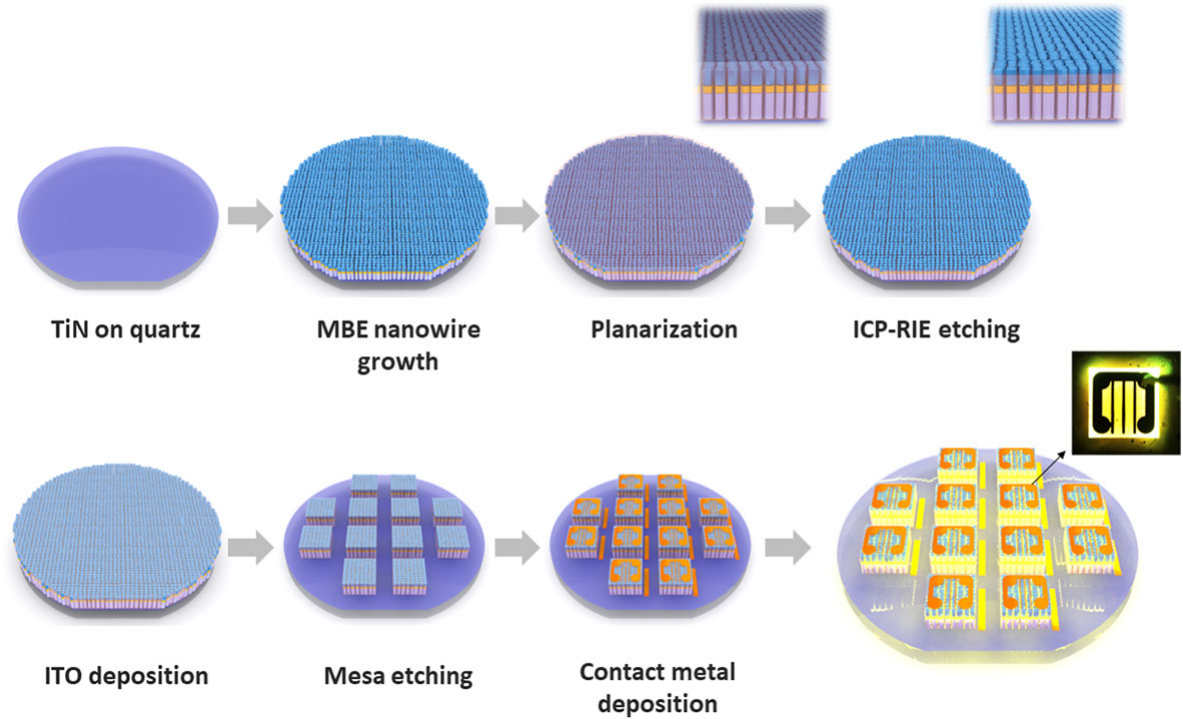
Fabrication steps for the nanowires on quartz LED

We incorporated the nanowires grown on quartz into LEDs. The fabrication steps are depicted in Fig. 4. The detailed fabrication steps are described in the “Methods” section.

**Fig. 5:**
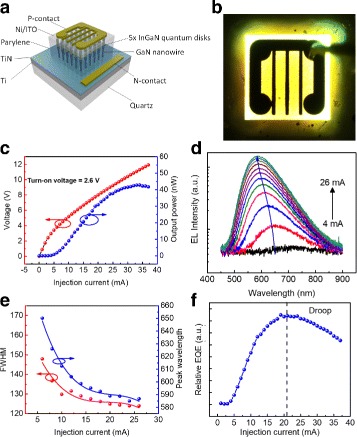
**a** Schematic of the fabricated LED device. **b** Optical photograph of the nanowires on quartz LED under forward bias. **c***L*-*I*-*V* characteristic of the LED. **d** Electroluminescence spectra of the LED under varying injection current. **e** Change of FWHM and peak wavelength position of the LED with increasing forward bias. **f** Relative external quantum efficiency of the LED, showing efficiency droop at higher injection current due to current crowding and junction heating

The LED structure depicted in Fig. 5a consists of the following layers: the Ni/Au contact pad, Ni/ITO transparent current spreading layer, GaN nanowires with five InGaN quantum disks embedded inside dielectric filling material (parylene C), and a bottom TiN/Ti interlayer. The bottom TiN/Ti interlayer acts as a translucent contact layer.

Figure 5 shows the electrical characterization results of the a 500 *μm*×500 *μm*-sized nanowire-on-quartz device. The turn-on voltage, by linear extrapolation of the linear region of the *V*-*I* curve, was determined to be ~ 2.6 V. The turn-on resistance (~ 300 *Ω*) is higher than that of nanowire-based LED devices fabricated on silicon and metal platform primarily because of the limited conductivity of the thin TiN/Ti layer in combination with the spontaneous formation of insulating TiO_2_ layer [36]. When device transparency is non-critical, the turn-on resistance can be improved by depositing a thicker Ti interlayer before growth. The light output power shown in the result of the *L*-*I* measurement is relatively low, as only light emitted normal to the device plane is collected. The light emission from the device in Fig. 5b shows that part of the light emitted by the device couples into the surrounding quartz substrate area and is partially backscattered normal to the substrate plane, resulting in low light extraction efficiency. However, this result also highlights the possibility of using the nanowire-on-quartz LED as the foundation for an all-optical circuit on a glass platform by carefully engineering the coupling and guiding of photons inside the quartz substrate.

Electroluminescence (EL) measurement results in Fig. 5d, e show a broad emission linewidth of above 120 nm. The electroluminescence peak agrees well with the room temperature *μ*-PL measurement. At a low injection current density approximately at turn-on, the LED exhibits a broad spectral emission near the red wavelength. With increasing injection current, the spectrum blue-shifts from 650 nm towards 590 nm thus realizing on-chip tuning across red-amber-yellow color regime. The blue-shift in peak wavelength is related to progressive band filling effect where at a high injection current electron starts filling higher energy state and recombine, resulting in emission at shorter peak wavelength. At higher injection current, the blue-shifting of the peak wavelength is saturated, due to competition between blue-shifting and red-shifting caused by the increase in junction temperature. Using a quantum-disk-in-nanowire structure, the polarization field is reduced through strain relief, thus enabling the realization of a yellow LED device which is challenging to achieve with a planar quantum well-based device.

**Fig. 6:**
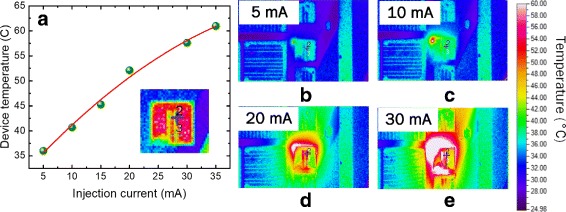
Device temperature measurement using the OptoTherm infrared camera. **a** Change of device temperature with increasing injection current. Inset shows infrared image of the device structure under zero bias and adjusted color bar. The measurement point is indicated by the number 2 and the purple cross. Infrared image corresponding to the temperature of the device and surrounding area at injection current of **b** 5, **c** 10, **d** 20, and **e** 30 mA. The results indicate that heat is concentrated in the area around the device

The relative external quantum efficiency (EQE) calculation shown in Fig. 5f shows that the quantum efficiency saturates at ~ 20 mA before it starts to decrease. This reduction in efficiency is caused by the combination of limited current spreading and junction heating effect, due to the low thermal diffusivity of quartz, resulting in heat buildup and efficiency roll-over within the device [37]. To investigate the junction heating within the device, an OptoTherm infrared camera was used to observe the device temperature under electrical injection directly. We performed temperature measurement on two different pixels, indicated by number 2 and 3 in the inset of Fig. 6a. However, for Fig. 6a, only measurement data from point number 2 is presented. At a current injection of 35 mA, the device temperature already exceeds 60 °C, which is noticeably higher compared to devices grown on top of silicon and metal. Figure 6b–d shows the heat distribution around the device at 5, 10, 20, and 30 mA. Under higher injection current, it can be seen that heat is not efficiently dissipated, but accumulates in the area around the device instead. Further detailed design of an efficient phonon transport medium, which is compatible with the current platform beyond this proof of concept demonstration, is required.

### Color Mixing Experiment

A CCT-tunable high-quality white light source plays an important role in consumer electronics, as it has been shown that the blue light component on the electronic display leads to suppression of melatonin, effectively interfering with human circadian rhythm [38, 39]. Capitalizing on the broadly tunable spectral characteristics of the device, we demonstrated a practical application of a widely CCT-tunable white light generation in a transmission configuration. We used the nanowire-on-quartz LED as the active widely tunable element with the red, green, and blue (RGB) laser diodes (LDs) as the secondary light sources. One advantage of using nanowire-based yellow light source for generating white light is the inherent broad emission, which leads to high color rendering index (CRI) value. By utilizing the yellow LED in conjunction with lasers, we were able to design a widely CCT-tunable white light. The arrangement of the color mixing setup is described as follows.

**Fig. 7:**
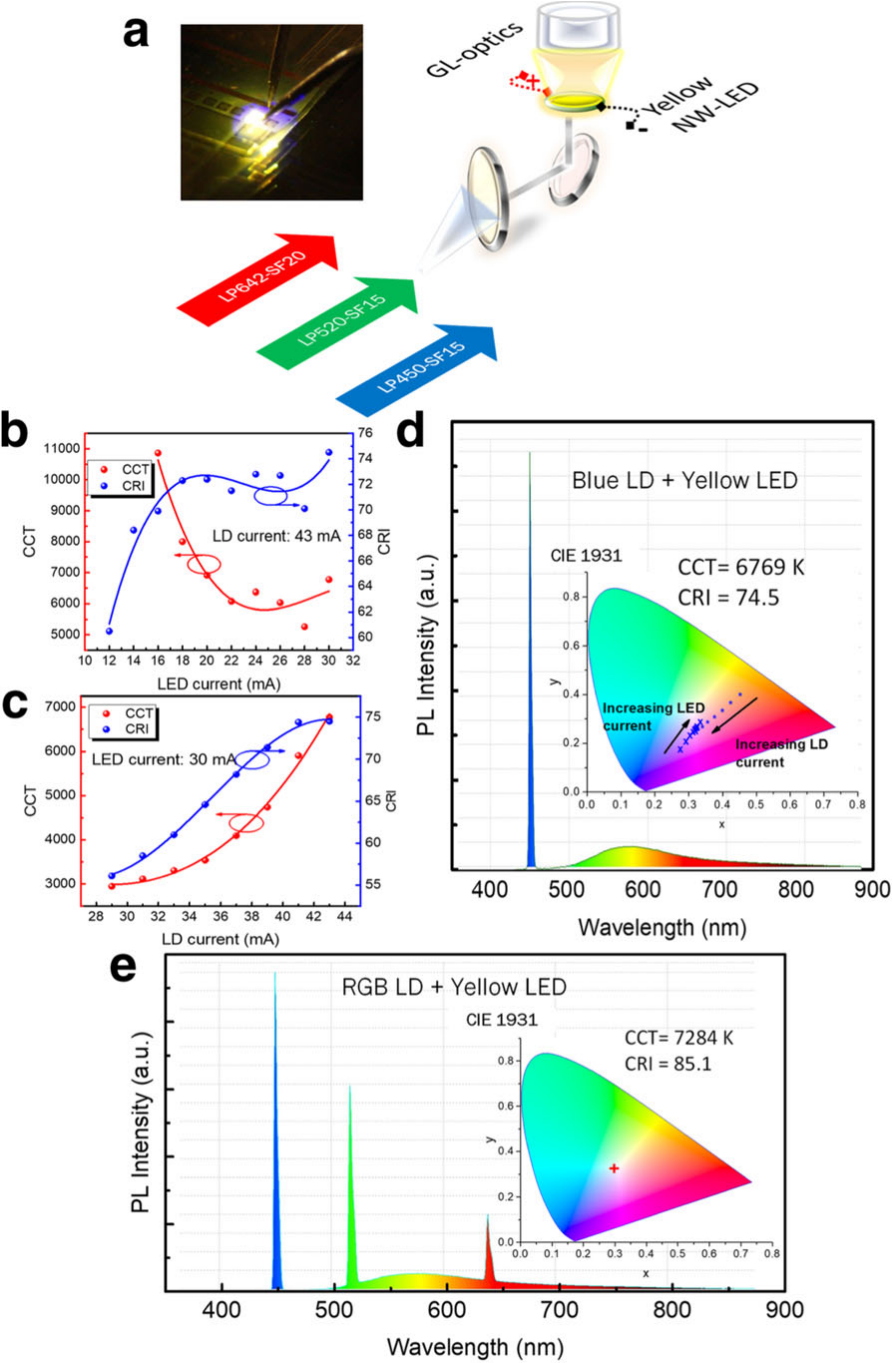
Color mixing experiment. **a** Color mixing experiment setup indicating the red, green, and blue LDs and the yellow nanowireon-quartz device. Inset shows optical photograph of LED under laser illumination. **b** Change in CCT and CRI with varying LED injection current. **c** Change in CCT and CRI with varying LD injection current. Wavelength spectrum and CIE 1931 map for a color mixing setup using **d** a blue LD with yellow nanowire-on-quartz LED and **e** RGB LDs with yellow nanowire-on-quartz LED

First, the outputs of the RGB LDs are combined using a Thorlabs three-channel wavelength combiner and collimated using a collimating lens. Next, the collimated beam is reflected using a 45° mirror onto the rear side of the nanowire-on-quartz LED and then passed through the top side of the LED. Finally, the detector is positioned directly above the nanowire-on-quartz LED to collect the resulting mixed color light. A schematic of the apparatus is shown in Fig. 7a. A GL Spectis 5.0 touch spectrometer was used to process the CRI and CCT values based on the International Commission on Illumination (CIE) 1931 standard.

In the first experiment, the beam from a blue LD was combined with the yellow light from the yellow LED. To obtain the highest CRI value possible, the bias currents of the LD and LED were initially varied, yielding a CRI value of 74.5 with a CCT value of 6769 K. This value is much higher than our previous result using a blue LD/YAG:Ce ^3+^ phosphor for white light generation [40]. To demonstrate the color tunability, either the LED or the LD bias was varied, starting from the bias value that produced the highest CRI. Figure 7b, c shows the effect of adjusting the bias current on the CRI and CCT values. We were able to tune the color temperature from 2800 K to over 7000 K while maintaining a CRI value above 55. Figure 7d shows the spectrum from the highest CRI achieved, with the inset showing the change in the CIE 1931 coordinate by varying the bias current. Further improvement to the CRI value was made using RGB LDs in conjunction with the yellow LED. When only the RGB LDs are utilized without the yellow LED spectrum component, we obtained a CRI value of 55.4. By incorporating the yellow spectrum component, we were able to obtain a high-quality white light with a CCT value of 7300 K and a CRI value of 85.1 (Fig. 7e), which is significantly higher.

By utilizing the nanowire-on-quartz LED in conjunction with laser diode system, we are able to design a widely CCT-tunable white light source while avoiding the issue of phosphor degradation [41]. By controlling the spectral characteristics of each wavelength individually, fine tuning of white light characteristics is possible. In addition, laser diode-based white light generation is more favorable compared to LED-based due to higher efficiency and potential cost advantage [42].

## Conclusions

In conclusion, we have demonstrated the growth of InGaN/GaN nanowires directly onto an amorphous quartz substrate using TiN/Ti interlayer and have fabricated LEDs based on the nanowire-on-quartz platform. By utilizing nanowire-based structure, we were able to grow highly crystalline III-nitride material on amorphous quartz. The nanowire-on-quartz LED enables the realization of an LED light source based on the scalable and economical substrate. The fabricated LED emits light at peak wavelength covering yellow-amber-red (peak wavelengths of 590 to 650 nm) with an FWHM of over 120 nm. Capitalizing on the broadly tunable spectral characteristics of the device, we demonstrated a practical generation of a widely tunable white light from 3000 to > 7000 K in a transmission configuration.

## References

[1] Bruni FJ (2015). Crystal growth of sapphire for substrates for high-brightness, light emitting diodes. Cryst Res Technol.

[2] Nabulsi F (2015) Implications for LEDs of the shift to large-diameter sapphire wafers. http://www.semiconductor-today.com/features/PDF/semiconductor-today-apr-may-2015-Implications-for.pdf. Accessed 10 Dec 2017.

[3] Asahi H, Iwata K, Tampo H, Kuroiwa R, Hiroki M, Asami K, Nakamura S, Gonda S (1999). Very strong photoluminescence emission from GaN grown on amorphous silica substrate by gas source MBE. J Cryst Growth.

[4] Kim JH, Holloway PH (2004). Room-temperature photoluminescence and electroluminescence properties of sputter-grown gallium nitride doped with europium. J Appl Phys.

[5] Bour DP, Nickel NM, Van de Walle CG, Kneissl MS, Krusor BS, Mei P, Johnson NM (2000). Polycrystalline nitride semiconductor light-emitting diodes fabricated on quartz substrates. Appl Phys Lett.

[6] Choi JH, Zoulkarneev A, Kim SI, Baik CW, Yang MH, Park SS, Suh H, Kim UJ, Bin Son H, Lee JS, Kim M, Kim JM, Kim K (2011). Nearly single-crystalline GaN light-emitting diodes on amorphous glass substrates. Nat Photonics.

[7] Choi JH, Cho EH, Lee YS, Shim MB, Ahn HY, Baik CW, Lee EH, Kim K, Kim TH, Kim S, Cho KS, Yoon J, Kim M, Hwang S (2014). Fully flexible GaN light-emitting diodes through nanovoid-mediated transfer. Adv Opt Mater.

[8] Shon JW, Ohta J, Ueno K, Kobayashi A, Fujioka H (2014). Fabrication of full-color InGaN-based light-emitting diodes on amorphous substrates by pulsed sputtering. Sci Rep.

[9] Bertness KA, Roshko A, Mansfield LM, Harvey TE, Sanford NA (2008). Mechanism for spontaneous growth of GaN nanowires with molecular beam epitaxy. J Cryst Growth.

[10] Hersee SD, Rishinaramangalam AK, Fairchild MN, Zhang L, Varangis P (2011). Threading defect elimination in GaN nanowires. J Mater Res.

[11] Guo W, Banerjee A, Bhattacharya P, Ooi BS (2011). InGaN/GaN disk-in-nanowire white light emitting diodes on (001) silicon. Appl Phys Lett.

[12] Lin HW, Lu YJ, Chen HY, Lee HM, Gwo S (2010). InGaN/GaN nanorod array white light-emitting diode. Appl Phys Lett.

[13] Connie AT, Nguyen HPT, Sadaf SM, Shih I, Mi Z (2014). Engineering the color rendering index of phosphor-free InGaN/(Al)GaN nanowire white light emitting diodes grown by molecular beam epitaxy. J Vac Sci Technol B Microelectron Nanometer Struct.

[14] Frost T, Jahangir S, Stark E, Deshpande S, Hazari A, Zhao C, Ooi BS, Bhattacharya P (2014). Monolithic electrically injected nanowire array edge-emitting laser on (001) silicon. Nano Lett.

[15] Jahangir S, Mandl M, Strassburg M, Bhattacharya P (2013). Molecular beam epitaxial growth and optical properties of red-emitting (*λ* = 650 nm) InGaN/GaN disks-in-nanowires on silicon. Appl Phys Lett.

[16] Jahangir S, Banerjee A, Bhattacharya P (2013). Carrier lifetimes in green emitting InGaN/GaN disks-in-nanowire and characteristics of green light emitting diodes. Phys Status Solidi (C) Curr Top Solid State Phys.

[17] Guo W, Zhang M, Banerjee A, Bhattacharya P (2010). Catalyst-free InGaN/GaN nanowire light emitting diodes grown on (001) silicon by molecular beam epitaxy. Nano Lett.

[18] Ristić J, Calleja E, Fernández-Garrido S, Cerutti L, Trampert A, Jahn U, Ploog KH (2008). On the mechanisms of spontaneous growth of III-nitride nanocolumns by plasma-assisted molecular beam epitaxy. J Cryst Growth.

[19] Zhao C, Ng TK, Wei N, Prabaswara A, Alias MS, Janjua B, Shen C, Ooi BS (2016). Facile formation of high-quality InGaN/GaN quantum-disks-in-nanowires on bulk-metal substrates for high-power light-emitters. Nano Lett.

[20] Sarwar AG, Carnevale SD, Yang F, Kent TF, Jamison JJ, McComb DW, Myers RC (2015). Semiconductor nanowire light-emitting diodes grown on metal: a direction toward large-scale fabrication of nanowire devices. Small.

[21] Wölz M, Hauswald C, Flissikowski T, Gotschke T, Fernández-Garrido S, Brandt O, Grahn HT, Geelhaar L, Riechert H (2015). Epitaxial growth of GaN nanowires with high structural perfection on a metallic TiN film. Nano Lett.

[22] Park Y, Jahangir S, Park Y, Bhattacharya P, Heo J (2015). InGaN/GaN nanowires grown on SiO_2 and light emitting diodes with low turn on voltages. Opt Express.

[23] Kumaresan V, Largeau L, Oehler F, Zhang H, Mauguin O, Glas F, Gogneau N, Tchernycheva M, Harmand JC (2016). Self-induced growth of vertical GaN nanowires on silica. Nanotechnology.

[24] Zhao S, Kibria MG, Wang Q, Nguyen HPT, Mi Z (2013). Growth of large-scale vertically aligned GaN nanowires and their heterostructures with high uniformity on SiO(x) by catalyst-free molecular beam epitaxy. Nanoscale.

[25] Wang W, Yang W, Wang H, Li G (2014). Epitaxial growth of GaN films on unconventional oxide substrates. J Mater Chem C.

[26] Carnevale SD, Yang J, Phillips PJ, Mills MJ, Myers RC (2011). Three-dimensional GaN/AlN nanowire heterostructures by separating nucleation and growth processes. Nano Lett.

[27] Jahangir S, Schimpke T, Strassburg M, Grossklaus KA, Millunchick JM, Bhattacharya P (2014). Red-emitting (lambda = 610 nm) In0.51Ga0.49N/GaN disk-in-nanowire light emitting diodes on silicon. IEEE J Quantum Electron.

[28] McCafferty E, Wightman JP (1999). An X-ray photoelectron spectroscopy sputter profile study of the native air-formed oxide film on titanium. Appl Surf Sci.

[29] Kiuchi M, Chayahara A (1994). Titanium nitride for transparent conductors. Appl Phys Lett.

[30] Uchida Y, Ito K, Tsukimoto S, Ikemoto Y, Hirata K, Shibata N, Murakami M (2006). Epitaxial growth of GaN layers on metallic TiN buffer layers. J Elec Materi.

[31] Chen NC, Lien WC, Shih CF, Chang PH, Wang TW, Wu MC (2006). Nitride light-emitting diodes grown on Si (111) using a TiN template. Appl Phys Lett.

[32] Tourbot G, Bougerol C, Glas F, Zagonel LF, Mahfoud Z, Meuret S, Gilet P, Kociak M, Gayral B, Daudin B (2012). Growth mechanism and properties of InGaN insertions in GaN nanowires. Nanotechnology.

[33] Besombes L, Kheng K, Marsal L, Mariette H (2001). Acoustic phonon broadening mechanism in single quantum dot emission. Phys Rev B.

[34] Guo W, Zhang M, Bhattacharya P, Heo J (2011). Auger recombination in III-nitride nanowires and its effect on nanowire light-emitting diode characteristics. Nano Lett.

[35] Nawaz M, Ahmad A (2012). A TCAD-based modeling of GaN/InGaN/Si solar cells. Semicond Sci Technol.

[36] Nihey F, Hongo H, Yudasaka M, Iijima S (2002). A top-gate carbon-nanotube field-effect transistor with a titanium-dioxide insulator. Jpn J Appl Phys.

[37] Feezell DF, Speck JS, DenBaars SP, Nakamura S (2013). Semipolar (2021) InGaN/GaN light-emitting diodes for high-efficiency solid-state lighting. J Disp Technol.

[38] Thapan K, Arendt J, Skene DJ (2001). An action spectrum for melatonin suppression: evidence for a novel non-rod, non-cone photoreceptor system in humans. J Physiol.

[39] Chang AM, Aeschbach D, Duffy JF, Czeisler CA (2015). Evening use of light-emitting eReaders negatively affects sleep, circadian timing, and next-morning alertness. Proc Natl Acad Sci.

[40] Lee C, Shen C, Oubei HM, Cantore M, Janjua B, Ng TK, Farrell RM, El-Desouki MM, Speck JS, Nakamura S, Ooi BS, DenBaars SP (2015). 2 Gbit/s data transmission from an unfiltered laser-based phosphor-converted white lighting communication system. Opt Express.

[41] Narendran N, Gu Y, Freyssinier JP, Yu H, Deng L (2004). Solid-state lighting: failure analysis of white LEDs. J Cryst Growth.

[42] Wierer JJ, Tsao JY (2015). Advantages of III-nitride laser diodes in solid-state lighting. Phys Status Solidi (A) Appl Mater Sci.

